# Supplementary material for: Content-rich biological network constructed by mining PubMed abstracts
Source: BMC Bioinformatics. 2004 Oct 8;5:147. doi: 10.1186/1471-2105-5-147 (PMC528731; doi:10.1186/1471-2105-5-147)
Supplement: Additional File 2 — The original results of the above study (non-essential files are deleted to keep the file size under the limit set by BMC bioinformatics). [file 1471-2105-5-147-S2.bz2 › chilibotAdditionalFile2/dip50/161ID8612709E566/html/ACVR1_ACVR2.html]

 


 **ACVR1** and **ACVR2** 
  
Found 63 abstracts in PubMed, retrieved 50.  
 

 What does Google say? 
 PDF only 
| .edu only 

---

**Interactive relationship** (e.g. stimulation, inhibition, etc)

**Neutral relationship**- OP 1 BMP 7 binds to BMPR II and ActR II  [ **ACVR2** ]  and forms complexes with BMPR IA and IB and ActR I  [ **ACVR1** ] .  Ref: 9094990 Am J Pathol, 1997
- In addition to these receptors, osteogenic protein 1 OP 1, also known as BMP 7 binds to activin type I receptor  [ **ACVR1** ]  ActR I  [ **ACVR1** ]  as well as activin type II receptors ActR II  [ **ACVR2** ]  and ActR IIB.  Ref: 8702914 J Biol Chem, 1996
- In the rat, activin acts via a family of activin receptor  [ **ACVR2** ]  ActR subunits that includes at least one type I ActRI or ALK 2  [ **ACVR1** ]  and two homologous type II IIA and IIB subunits.  Ref: 8593801 Endocrinology, 1996
- At E15, the expression of ActR II  [ **ACVR2** ]  mRNA was stronger than that of BMPR II in the spinal cord, followed in intensity by the expression of ActR I  [ **ACVR1** ] , ActR IB, BMPR IA, BMPR IB and TbetaR I mRNA.  Ref: 8854897 Cell Tissue Res, 1996

**Non-interactive relationship** (e.g. studied together, co-existance, homology, etc.)

- Activin A bound to ActR IB ALK 4 and ActR II  [ **ACVR2** ] , and BMP 7 bound to ActR I  [ **ACVR1** ]  ALK 2  [ **ACVR1** ] , BMP type I receptor BMPR I ALK 3, ActR II  [ **ACVR2** ]  and BMPR II.  Ref: 10574705 J Cell Sci, 1999
- BMP 6 strongly bound to activin receptor  [ **ACVR2** ]  like kinase ALK 2  [ **ACVR1** ]  also termed ActR I  [ **ACVR1** ] , together with type II receptors, i.e. BMP type II receptor BMPR II and activin type II receptor ActR II  [ **ACVR2** ] .  Ref: 10504300 J Cell Sci, 1999
- Both type I and type II activin receptor  [ **ACVR2** ]  ActR I  [ **ACVR1** ] , ActR IB and ActR II  [ **ACVR2** ]  mRNA expression was also detectable in the pheochromocytoma tissues.  Ref: 10810314 J Endocrinol, 2000
- To investigate the expression and function of specific Type I TGFbeta receptors during AV cushion transformation, we cloned and characterized the chicken homologues of two mammalian activin receptor  [ **ACVR2** ]  like kinases ALK, ALK2  [ **ACVR1** ]  and ALK5, and generated specific, polyclonal antibodies against the extracellular binding domains of each.  Ref: 10885742 Dev Biol, 2000
- A mechanistic explanation for increased pSmad1 5 staining during inflammation was provided by the upregulated expression of all the BMP type I receptors, i.e., activin receptor  [ **ACVR2** ]  like kinase ALK 2  [ **ACVR1** ] , ALK3, and ALK6, in the inflamed bronchial epithelial cells.  Ref: 12151307 Am J Respir Cell Mol Biol, 2002
- The type I receptors examined were the TGF beta type I receptor TbetaR I, activin type I receptor  [ **ACVR1** ]  ActR I  [ **ACVR1** ] , activin type IB receptor ActR IB, bone BMP type IA receptor BMPR IA, BMP type IB receptor BMPR IB, and activin receptor  [ **ACVR2** ]  like kinase 1 ALK 1.  Ref: 10321529 Acta Ophthalmol Scand, 1999
- The ActR I  [ **ACVR1** ]  activin receptor  [ **ACVR2** ]  protein is expressed in notochord, lens placode and pituitary primordium cells in the mouse embryo.  Ref: 10704880 Mech Dev, 2000
- Molecular cloning has yielded genes encoding two type II receptors ActRII  [ **ACVR2** ]  and ActRIIB and at least two type I receptors ALK2  [ **ACVR1** ]  and ALK4.  Ref: 8971337 Biochem Soc Symp, 1996
- RESULTS Activin A and receptors ActR I  [ **ACVR1** ] , ActR IB, ActR II  [ **ACVR2** ]  are transcribed in corneal fibroblasts.  Ref: 11773015 Invest Ophthalmol Vis Sci, 2002
- To explore the molecular cascade of odontoblast differentiation, Northern blot analyses and reverse transcriptase polymerase chain reaction were here used to investigate the expression patterns of the genes for TGF beta superfamily members TGF beta 1, namely bone morphogenetic protein BMP 4, BMP 7, activin beta A and activin beta B, and their type I and type II receptors, namely activin receptor  [ **ACVR2** ]  like kinase ALK 2  [ **ACVR1** ]  ActR I  [ **ACVR1** ] , ALK 3 BMPR IA, ALK 4 ActR IB, ALK 5 T beta R I, BMPR II and T beta R II, during differentiation of pulp cells into preodontoblasts in bovine adult pulp cell culture.  Ref: 9296267 Arch Oral Biol, 1997
- Activin receptor  [ **ACVR2** ]  like kinase 2  [ **ACVR1** ]  can mediate atrioventricular cushion transformation.  Ref: 10885742 Dev Biol, 2000
- The presence of inhibin subunits alpha, betaA and betaB, activin binding protein follistatin and activin receptors ActR I  [ **ACVR1** ] , ActR IB, ActR II  [ **ACVR2** ]  and ActR IIB mRNA transcripts in bovine oocytes and preimplantation embryos was examined by reverse transcription polymerase chain reaction.  Ref: 11596877 Reprod Fertil Dev, 1998
- The recently cloned bone morphogenetic factor receptor type II BMPR II was compared with the ActR II  [ **ACVR2** ]  and several type I receptors including ActR I  [ **ACVR1** ] , ActR IB, BMPR IA, BMPR IB and TbetaR I.  Ref: 8854897 Cell Tissue Res, 1996
- In Xenopus embryo, Smad8 is able to transcriptionally activate a subset of mesoderm target genes similar to those induced by the receptor serine kinase, activin receptor  [ **ACVR2** ]  like kinase ALK 2  [ **ACVR1** ] .  Ref: 9371779 Proc Natl Acad Sci U S A, 1997
- Primers specific for two type I and two type II activin receptors ActR I  [ **ACVR1** ] , ActR IB, ActR II  [ **ACVR2** ] , and ActR IIB were used in polymerase chain reaction PCR to amplify cDNAs prepared from granulosa luteal cells, placental tissues and isolated trophoblast cells.  Ref: 10210281 Life Sci, 1999
- The effect of activin is most likely mediated through specific receptors as mRNAs encoding several forms of activin receptors, namely ActR I  [ **ACVR1** ] , ActR IB, ActR II  [ **ACVR2** ]  and ActR IIB are found in the preovulatory follicles as well as in cultured granulosa luteal cells.  Ref: 8836491 Biol Signals< MedlineTA>Biol Signals, 1999
- In the adult brain, mRNA for BMPR II was expressed in different patterns together with ActR II  [ **ACVR2** ]  and ActR I  [ **ACVR1** ] .  Ref: 8854897 Cell Tissue Res, 1996
- In particular, the brain is expressing high levels of bone morphogenetic protein receptor type II BMPR II, activin receptor  [ **ACVR2** ]  type I  [ **ACVR1** ]  ActR I  [ **ACVR1** ] , and activin receptor  [ **ACVR2** ]  type IIA ActR II  [ **ACVR2** ] .  Ref: 9469567 J Neurosci Res, 1998
- Receptor Alk2  [ **ACVR1** ]  protein levels were significantly elevated in T PE placentae P=0.0006, Mann Whitney U test, ActRIIB levels were significantly lower in PT PE placentae P=0.01 and ActRII  [ **ACVR2** ]  levels were significantly lower in PT PE choriodecidua P=0.0002 compared with controls.  Ref: 11572790 J Endocrinol, 2001
- We studied the expression of BMPR IA, IB, and II, ActR I  [ **ACVR1** ] , ActR II  [ **ACVR2** ] , and OP 1 BMP 7 by immunohistochemistry in ossified ligament tissues of patients with OPLL and control ligament tissues from patients with cervical disc herniation.  Ref: 9094990 Am J Pathol, 1997
- BMPR I group and those of activin receptor  [ **ACVR2** ]  like kinase ALK 1 and ALK 2  [ **ACVR1** ]  ALK 1 group induced alkaline phosphatase activity in C2C12 cells.  Ref: 10564272 Mol Biol Cell, 1999
- This study demonstrated that ActR I  [ **ACVR1** ] , ActR 1B and ActR II  [ **ACVR2** ]  are expressed on human fetal lung fibroblast.  Ref: 11261590 Life Sci, 2000
- ActR II  [ **ACVR2** ] , ActR I  [ **ACVR1** ] , ActR IB and, weakly, TbetaR I were all expressed in the dentate gyrus.  Ref: 8854897 Cell Tissue Res, 1996
- Furthermore, the effects of ALK 2  [ **ACVR1** ] \* signalling are at odds with it behaving as an activin receptor  [ **ACVR2** ]  in the early Xenopus embryo.  Ref: 9367435 Development, 1997
- these cells were found to express mRNA for BMP receptors including, ActR I  [ **ACVR1** ] , BMPR IA, ActR II  [ **ACVR2** ] , ActR IIB, and BMPR II.  Ref: 11786084 Kidney Int, 2002
- When coexpressed with type I activin receptor  [ **ACVR2** ]  ActR I  [ **ACVR1** ]  or ActR IB in concert with type II receptor ActR II  [ **ACVR2** ] , Smad7 efficiently reduced an early transcriptional response mediated by ActR I  [ **ACVR1** ]  but had only a minimal effect on the response mediated by ActR IB.  Ref: 10828018 Blood, 2000
- BMP type II receptor BMPR II is a type II serine threonine kinase receptor, which transduces signals for BMPs through heteromeric complexes with type I receptors, including activin receptor  [ **ACVR2** ]  like kinase 2  [ **ACVR1** ]  ALK2  [ **ACVR1** ] , ALK3 BMPR IA, and ALK6 BMPR IB.  Ref: 10772805 Dev Biol, 2000
- Furthermore, transfection with wild type activin receptor  [ **ACVR2** ]  type I  [ **ACVR1** ]  results in activin mediated suppression of cell proliferation.  Ref: 12364468 J Clin Endocrinol Metab, 2002
- We have also explored if there are associated changes in activin receptor  [ **ACVR2** ]  Alk2  [ **ACVR1** ] , ActRII  [ **ACVR2** ]  and ActRIIB in these tissues.  Ref: 11572790 J Endocrinol, 2001
- PCR products with the expected sizes for ActR I  [ **ACVR1** ] , ActR IB, ActR II  [ **ACVR2** ] , and ActR IIB mRNAs were detected in freshly dissociated and 5 day cultured granulosa luteal cells.  Ref: 10210281 Life Sci, 1999
- Constitutively active forms of BMP type I receptors BMPR IA and BMPR IB and those of activin receptor  [ **ACVR2** ]  like kinase ALK 1 and ALK 2  [ **ACVR1** ]  maintained differentiation of chondrocytes in the presence of retinoic acid.  Ref: 12565908 Biochem Biophys Res Commun, 2003
- Three lysines, Lys 431, Lys 440, and Lys 441 in p50 were all acetylated in vitro, and a sequence similarity among p50, p53, Tat, and activin receptor  [ **ACVR2** ]  type I  [ **ACVR1** ]  on these particular lysines was observed.  Ref: 11739381 J Biol Chem, 2002
- Using the cDNA clones of activin receptor  [ **ACVR2** ]  type I  [ **ACVR1** ]  and II, we have shown that both receptors are expressed in this cultured cells.  Ref: 7594884 Nippon Sanka Fujinka Gakkai Zasshi, 1995
- We then investigated whether the sensitivity of the early long bone rudiments to OP 1 correlated with high expression of the OP 1 binding type I serine threonine kinase receptors activin receptor  [ **ACVR2** ]  like kinase ALK 2  [ **ACVR1** ]  ActR I  [ **ACVR1** ] , ALK 3 BMPR IA or ALK 6 BMPR IB at this early stage.  Ref: 10705576 Growth Factors, 2000
- Analysis in native tissues from 12 normal thyroids, 16 goiters, and 5 Graves thyroids demonstrated mRNA expression of the activin subunit betaA, TGFbeta receptor type I and II, activin receptors type I receptors alk2  [ **ACVR1** ]  and alk4, and activin type II receptors actRII  [ **ACVR2** ]  and actRIIb.  Ref: 11069203 Horm Metab Res, 2000
- Furthermore, bovine oocytes and embryos express the activin receptors mainly by the combination of ActR I  [ **ACVR1** ]  and either ActR II  [ **ACVR2** ]  or ActR IIB.  Ref: 11596877 Reprod Fertil Dev, 1998
- There were no apparent differences in the distribution of the beta A subunit and receptors Alk2  [ **ACVR1** ] , ActRII  [ **ACVR2** ]  and ActRIIB between control and preeclamptic tissues.  Ref: 11572790 J Endocrinol, 2001
- Activin receptor  [ **ACVR2** ]  type I  [ **ACVR1** ]  and II mRNA amplified products were found in virtually all the surface epithelial samples and ovarian tumors.  Ref: 9570994 Gynecol Oncol, 1998
- Activin receptor  [ **ACVR2** ]  like kinase ALK 1, ALK 2  [ **ACVR1** ] , ALK 3, and ALK 6 were able to reproduce the hypertrophic maturation of chondrocytes induced by BMP 2.  Ref: 12082094 J Biol Chem, 2002
- In this study, we found the expression of activin type I receptors ActR I  [ **ACVR1** ]  and ActR IB and type II receptor ActR II  [ **ACVR2** ]  on human fetal lung fibroblasts HFL 1 by RT PCR and immunocytochemistry.  Ref: 11261590 Life Sci, 2000
- Renal expression of the high affinity BMP type II receptor and the type I receptor Alk2  [ **ACVR1** ]  activin receptor  [ **ACVR2** ]  like kinase 2  [ **ACVR1** ]  decreased.  Ref: 11675415 J Am Soc Nephrol, 2001
